# Supplementary material for: Psychophysiological Interactions in a Visual Checkerboard Task: Reproducibility, Reliability, and the Effects of Deconvolution
Source: Front Neurosci. 2017 Oct 17;11:573. doi: 10.3389/fnins.2017.00573 (PMC5651039; doi:10.3389/fnins.2017.00573)

**Supplementary materials for “Psychophysiological Interactions in a Visual Checkerboard Task: Reproducibility, Reliability, and the Effects of Deconvolution”**

Xin Di (*synge.x.d@gmail.com*) and Bharat B. Biswal

This file contains supporting information for the paper “Psychophysiological Interactions in a Visual Checkerboard Task: Reproducibility, Reliability, and the Effects of Deconvolution”. This includes one supporting table and two supporting figures.

**Table S1** Voxel-wise psychophysiological interaction (PPI) results of the thalamus seed for the two TR (repetition time) runs and two methods.

| Label | Voxels | p (FDR) | Peak MNI coordinate | | | Peak t |
| --- | --- | --- | --- | --- | --- | --- |
|  |  |  | x | y | z |  |
| TR = 645 ms, with deconvolution | | | | | | |
| Middle cingulate gyrus | 54 | 0.010635 | -6 | 8 | 32 | 5.031316 |
| R. thalamus | 58 | 0.010635 | 6 | -7 | 2 | 4.569748 |
| L. thalamus | 33 | 0.026576 | -6 | -13 | 5 | 4.447518 |
| L. fusiform gyrus | 56 | 0.010635 | -30 | -70 | -13 | 4.32583 |
| L. anterior insula | 35 | 0.024985 | -30 | 14 | 5 | 4.087661 |
| R. anterior insula | 48 | 0.013306 | 33 | 26 | 5 | 4.072107 |
| R. basal ganglia | 39 | 0.01994 | 21 | 11 | -1 | 4.049067 |
| R. fusiform gyrus | 42 | 0.018151 | 27 | -43 | -19 | 3.90397 |
| TR = 1400 ms, with deconvolution | | | | | | |
| n.s. |  |  |  |  |  |  |
| TR = 645 ms, without deconvolution | | | | | | |
| R. occipital pole | 78 | 3.55E-05 | 9 | -88 | 14 | -5.49644 |
| L. occipital pole | 49 | 0.000628 | -21 | -100 | 5 | -4.8916 |
| TR = 1400 ms, Without deconvolution | | | | | | |
| n.s. |  |  |  |  |  |  |

FDR, False discovery rate; MNI, Montreal Neurological Institute; L, left; R, right; n.s. not significant.

**Figure S1** Maps of intraclass correlations of psychophysiological interactions (PPI) effects between the two TR runs for the left middle occipital gyrus (LMOG) and right middle occipital gyrus (RMOG) seeds and two PPI methods. Only positive effects are shown.


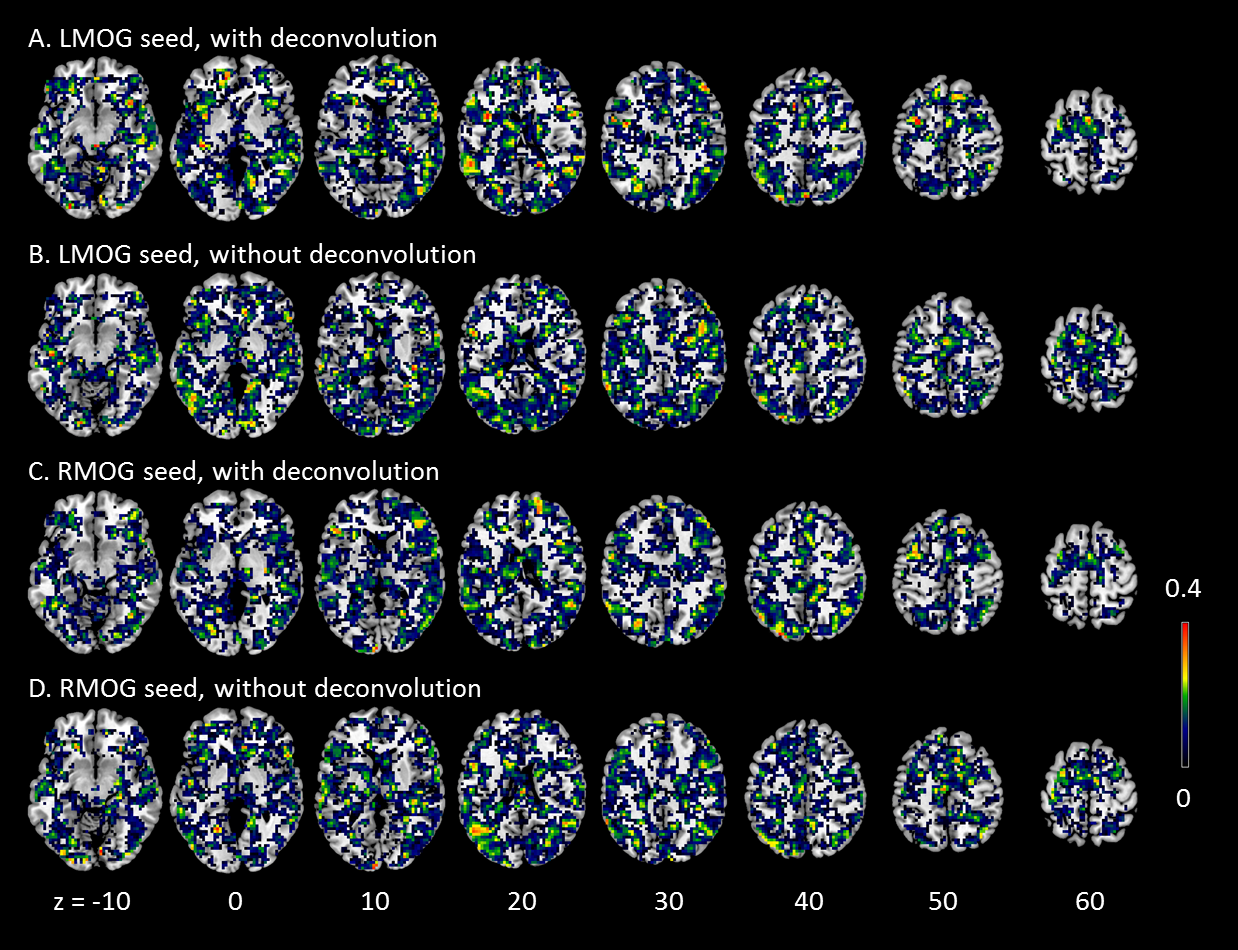


**Figure S2** Matrices of intraclass correlations of psychophysiological interaction effects between the two TR runs among 20 visual thalamic and cortical regions.


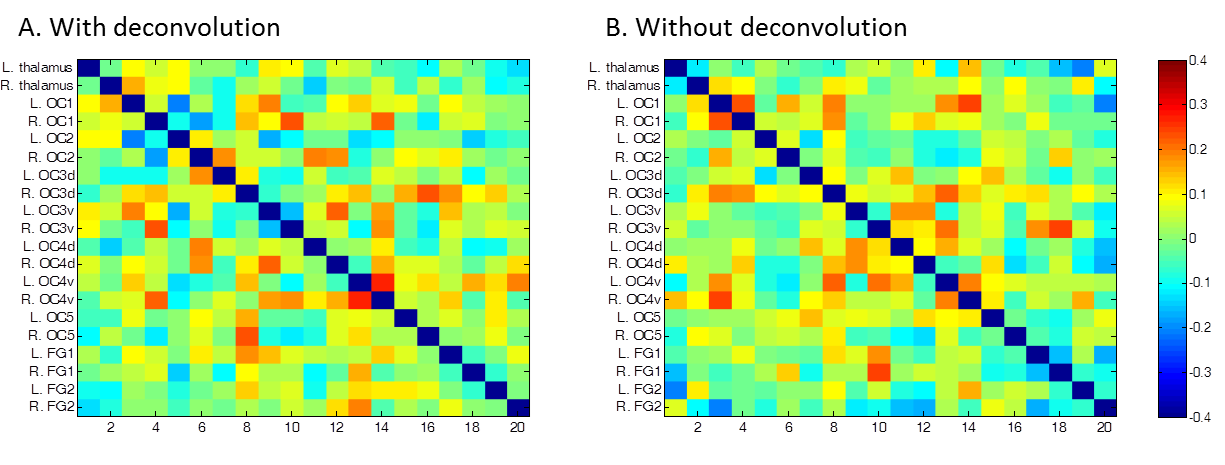

Supplement: Supplementary file 1 [file DataSheet1.DOCX]
